# Biased technical change in hospital care and the demand for physicians

**DOI:** 10.1186/s12960-020-00500-z

**Published:** 2020-08-20

**Authors:** Jos L. T. Blank, Thomas K. Niaounakis, Vivian G. Valdmanis

**Affiliations:** 1 Institute of Public Sector Efficiency Studies, Delft University of Technology, P.O. Box 5015, 2600 GA Delft, the Netherlands; 2 Western Michigan University, Kalamazoo, MI USA

**Keywords:** Labour demand, Medical specialists, Technological developments, Productivity

## Abstract

**Background:**

The development of labour productivity is relevant for accurately planning future staffing requirements, especially in sectors where technological developments may drive labour substitution. The present study investigates how labour productivity has developed across Dutch medical specialists (2007–2017) and discusses its implications for workforce planning, also in relation to the existing literature.

**Methods:**

A regression model is developed in which the number of full-time equivalents is related to production (admissions), hospital characteristics and a trend parameter. The trend parameter captures the average annual change in the number of full-time equivalents per production output and is a measure for labour productivity. The model is applied to a micro-data set of Dutch hospitals in the period 2007–2017 across 24 different specialties using regression methods.

**Results:**

The results indicate an increase in the number of full-time equivalents per admission has increased for most specialisms and that labour productivity has thus decreased. However, there is considerable heterogeneity and uncertainty across different specialisms.

**Conclusions:**

The results amplify the issue of medical personnel shortages driven by the growing demand for health care. The research outcomes are linked to the existing literature regarding physicians’ productivity. Changes in accountability such as using relative value units, incentive payments, use of staff and mid-level providers, and technology have been discussed, and some consensus has been reached.

## Introduction

In order to meet the future demand for care, it is important to train enough medical care staff in good time. Factors that influence the demand for medical staff include ageing population with several co-morbidities, patients’ chronic and acute care needs and on-going technological progress [1, 2]. Besides direct medical conditions, socio-economic factors such as income, education, and gender should also be considered. Socioeconomic factors are relevant since lower income or education groups may have poor health habits such as smoking, poor diet, and living in less desirable neighbourhoods that may have poorer environmental quality. As these factors may increase the demand for healthcare services including hospital services, this may add a burden on hospitals’ resources including medical specialists. Given these factors, labour productivity and ensuring enough physicians are available are relevant for policy-makers and hospital decision-makers.

The Advisory Committee on Medical Manpower Planning in the Netherlands (*Capaciteitsorgaan*) compiles three-yearly estimates of the future healthcare professional capacity required to meet demand for hospital services. In addition to the development of healthcare demand and changes in the labour market (outflow, retirement), an important parameter of interest is labour productivity. Health care innovations and new treatment methods mean that, with time, fewer staff (or possibly more staff) will be needed per patient treated than are currently required. Hence, there is a need to measure productivity trends in the health care sector. To address this need, we have provided productivity trend estimates across different medical specialists in The Netherlands for the period 2007–2017, regressing FTEs per specialism on production and a trend reflecting technical change, using detailed hospital data. The trend not only includes the influence of technological developments, but also social developments such as the growing preference of medical staff to work part-time, and a changing case mix of patients.

## Literature review

Labour productivity is an important topic to analyse for several reasons. Firstly, productivity is needed to measure long-term stability in a health system; secondly, the labour force is the most important resource in any health care system; and thirdly, in 2014, there were 21 million people employed in the health care sector within the EU15 (the EU member states prior to 1 May 2004), mostly in hospitals and ambulatory care settings [1], making it an important sector. Hofmarcher et al. [1] further argue that because of the variation in employment among these countries, there is a call for policy-makers and hospital decision-makers to better utilise the health care work force. Early publications on productivity suggest that physician productivity in terms of number of visits has been declining, suggesting that projections of a physician surplus have been overstated [3]. These authors found that reducing the patient workload of physicians in the United States of America by a few hours per week would eliminate this surplus. Reuben et al. [4] assessed the factors affecting US physician productivity and found that even with an ageing population and a higher case mix among patients, physicians enhanced productivity. Given their findings, Reuben et al. [4] suggested that health policy be directed at increasing the use of mid-level practitioners. Greenberg and Cultice [5] used what they claimed to be a better data source (Health Resources and Services Administration data) to forecast the number of physicians needed. By analysing eighteen medical specialties over several settings including surgery visits, accident and emergency (A&E) visits, hospital outpatient visits, inpatient hospital stays, surgeries, and long-term care and using the number of minutes per service as an output, they found that by 2020 there would be a total need of 772 500 physicians in the United States of America, broken down into 254 200 primary care physicians, 482 400 specialists, and 35 900 doctors of osteopathy. These authors further demonstrated that the percentage of primary care physicians in the United States of America would not change much over time and that productivity growth by specialty varied from 7% for urologists to 22% for pathologists.

Goodman [6] echoed this sentiment by showing that the need was not for more physicians but for improved productivity. He also suggested that physicians should be linked to a well-defined patient population (either demographically or by specialty) and that, since 1984, there was no systematic difference in known processes of care or health outcomes. Interestingly, Goodman [6] found that very low levels of specialists led to poorer outcomes but even a high density of specialists was not associated with better outcomes, emphasising the need for having the right mix of specialty care.

Moving beyond physician-patient care as a productivity measure, Andreae et al. [7] found that incentive-based physician compensation enhanced productivity including clinical and scholarly productivity, quality of medical education, and faculty satisfaction, all of which account for physician time in an academic medical setting. Arguments that a compensation package based on productivity would mimic the fee-for-service payment schemes of the past which rewarded physicians as a function of the number of treatments and tests provided often overestimate actual physician productivity. Storfa and Wilson [8] also made a similar argument in favour of incentive payments based on relative value units (RVUs) which resemble health outcomes rather than healthcare outputs. In addition to incentive payments, there needs to be a better balance between workload and productivity because of the specialisation of service delivery. Also, physicians should not be penalised for working in a low patient volume setting but whose services are needed nonetheless, and physicians also spend time in other non-billable activities including education and research. These authors further argue that it is not only physician productivity, but also efficiency at system level including the organisation and administration.

Whereas it has been demonstrated in the literature that the use of mid-level practitioners enhances physician productivity, Park and Jones [9] found that in the increased use of hospitalists (physicians who solely work with inpatients), there was an increase in primary care providers’ surgery-visit productivity by approximately 10%. This increase in surgery visits also translated to fewer hospital visits, primarily due to higher quality primary care. This finding corresponds with Mechanic et al.’s [10] study showing that the number of minutes spent with patients in surgery visits increased. This increase in time spent with patients in the outpatient setting will be enhanced with more hospitalists. Fewer hospitalisations may not necessarily correspond to changes in A&E visits. Joseph et al. [11] used multivariate analysis to show that physician productivity in A&Es at three community hospitals decreased as the shift progressed. In other words, productivity was higher earlier than later in the shift, indicating decreasing marginal productivity and calling for better physician staffing within the A&E department.

Cheriff et al. [12] focused on the specific use of electronic health records (EHRs). As physicians used this system longer, physician productivity increased at a statistically significant rate in terms of average monthly visits and also in 12 weighted RVUs. These differences were measured between physicians who fully adopted EHRs and physician who did not adopt this technology, operating in a multi-specialty medical group.

Physician productivity has also been measured in Norway following health care reforms there. Rather than analysing changes at macro level, Johannessen et al. [13] analysed the effects of the reforms on the labour levels between 2001 and 2013. They found that that in general, the number of patients treated increased by 47% and DRG scores increased by 35%, indicating that more and sicker patients were being treated. However, there were no significant increases in physician productivity but rather in more registered nurses and more secretaries per FTE physician. Hence, these authors concluded that more support staff were associated with predicting physician productivity.

To sum up physician productivity in general, Fry and Nicols [14] identified four steps to improving physician productivity and three barriers to this. These barriers included documentation, laboratory turnaround times, and challenges with EHR, particularly for physicians not used to this system, as documented by Cheriff et al. [12]. The four enhancements include leadership between physicians and administration, measuring productivity using more than three measures, ensuring incentive alignment, and identifying outcomes. Note that some of these enhancing measures have been discussed in earlier research. These range from using more measures, including services that are not billable [8], incentive pay [7, 8], to positive productivity changes and identifying outcomes in terms of RVUs rather than the number of treatments and tests ordered as under fee-for-service [7].

Brownlee et al. [15] analysed labour productivity in the United States of America. Because of supply-sensitive tendencies among providers, too many hospitalisations and tests are ordered simply because these resources are available. These supply-sensitive tendencies mean productivity is deemed lower. These authors advocate assessing the value of services and tests and reducing the poor organisation which may include physicians being excessively burdened by paperwork. Because of these deficiencies in the US health care services, increasing the number of specialist physicians would lead to even more inefficiency [15].

Conversely, Dall et al. [16] argue along the lines of Hofmarcher et al. [1] that due to an ageing population and its concomitant disease burden, more rather than fewer specialists will be needed to provide high-quality and effective care. This argument is based on the finding that long waiting times for specialist care may lead to deteriorating health conditions and lower well-being. Assessing the needs in all 50 states based on a weighted survey of three million patients, they found that there was a need for 14% more primary care providers, 20% more cardiologists, and 31% more vascular surgeons. It was also suggested that neurologists will be used in a more consultative role; therefore, a team of specialists will be required for full treatment of patients.

Interestingly, Blank and van Hulst [2] differentiate between factor technical change (FTC) and total factor productivity (TFP). FTC is due to changes from technical changes controlling for output and input prices, whereas TFP measures changes due to efficiency change (movement along the frontier) and technological change (movement to another frontier). The findings reported there included that productivity increased 28% per year, but that technological change was attributed to input-bias with the latter defined as more is not necessarily better. This phenomenon arises because some input measures can be manipulated that lead to biased outcome assessment. Note the similarity between input bias as the supply sensitivity referred to by Brownlee et al. [15].

These studies reflect physician productivity with different conclusions. Brownlee et al. [15] argue there are too many specialists; Dall et al. [16] present a counter argument that there are too few specialists. We attempt to resolve these contradictions by assessing the productivity of specialists and not just a perceived need measured by waiting times. Further, we focus on the specialists in order to address the concern raised by Hofmarcher et al. [1] that maintaining a sustainable hospital system, allocating specialists by productivity would better utilise these important resources.

## Methods

To calculate labour productivity, we use a model in which the number of FTEs per specialty is related to production, the type of hospital (to control for case mix differences) and a trend parameter. This model is a mathematical representation of the relationship between FTE medical specialists on the one hand and the production and technical changes on the other [2]. In the present case, this means that a separate equation is included for each distinguished medical specialty. In formula form:

1 $$ \ln \left(\mathrm{ft}{\mathrm{e}}_s\right)={a}_{0s}+\sum \limits_m{b}_{ms}\ln \left({y}_{ms}\right)+{h}_st+{d}_{1s}{\mathrm{gen}}_s+{d}_{2s}\mathrm{du}{\mathrm{m}}_s $$

fte_*s*_ = full-time jobs in specialty s

*y*_*ms*_ = type m production in specialty s

*t* = time

gen = dummy general hospital

dum = data correction dummy

The equation also contains two dummy variables. The first dummy variable indicates whether it is a general hospital (i.e. no teaching, categorical or a top clinical hospital). In fact, this variable provides a rough correction for differences in case mix between types of hospitals as well as different demands on physician time including teaching and research. The second dummy variable is included to estimate the correction for a specific data problem occurring in 2013. Separate parameters are therefore estimated for each specialty. Furthermore, we impose the restriction of homogeneity of degree 1 on the parameters *b*_*ms*_:

$$ \sum \limits_m{b}_{ms}=1 $$

This restriction implies that if all product types grow by 1%, the use of medical specialists automatically grows by 1%. This restriction comes from the assumption that no (dis)economies of scale exist.

The parameter value of the trend (*h*_*s*_) indicates the average annual increase or decrease in the use of staff at a given level of production. In fact, this measure is the mirror image of labour productivity. More FTEs needed given production levels implies a decline in labour productivity (and vice versa).

The production of a specialty is measured here by the number of admissions for each specialty and the total number of admissions for the entire hospital. This last production indicator is important for the supporting specialties, which in fact do not have their ‘own’ patients. Furthermore, the estimates consider the distinction between general hospitals and top clinical hospitals and teaching hospitals.

The use of microdata provides major advantages, allowing technical changes to be statistically assessed from year to year. This makes it possible to see whether this is a general trend or whether the outcome is influenced by all kinds of specific developments, i.e. input-biased. Models using macro-level data cannot provide these insights. The use of microdata also provides a more accurate picture of the production process, because many more observations can be used.

## Resources and data

The data were provided by Dutch Hospital Data (DHD). The variables related to the production of hospitals come from the National Basic Registration of Hospital Care (LBZ). Data on personnel are derived from the Survey Annual Figures of Hospitals (EJZ). Not all hospitals participate in the EJZ every year, which varies the coverage rate of the EJZ per year. Finally, the analysis also uses several descriptive variables at hospital level. These figures also come from the EJZ. As noted, the unit of analysis is a specialty. A total of 35 specialties are identified. Of these, 24 are included in the study. The remaining eleven specialties were excluded from the analysis due to the limited number of hospitals providing these specialties and the low number of patients involved.

The input of personnel is expressed in FTEs and is derived from an annual survey of hospitals (EJZ). Two indicators are available: the number of FTEs per year and per specialty and the total number of FTEs.

The production per specialty is based on the LBZ. We measure production per specialty based on the number of outpatient and clinical admissions within each specialty and the number of outpatient and clinical admissions for each hospital. For the second variable, the relevant indicators are aggregated per hospital as a sum of all specialties.

The database is made up of 15 424 observations with 2640 originating from teaching hospitals. The remaining observations originate from general, top clinical, or specialist hospitals. The coverage rate of teaching hospitals in the data files provided is 100%, but for the other hospitals, individual consent was required. Furthermore, observations often proved unusable because at least one or more of the indicators requested were not completed. In the end, 7063 observations were used. Each observation contains information about the input of personnel and production for a specialty within a hospital at a given year. For each hospital listed in the file, one observation per specialty per year is available, provided that the hospital has the specialty in question.

## Estimation and results

**Fig. 1:**
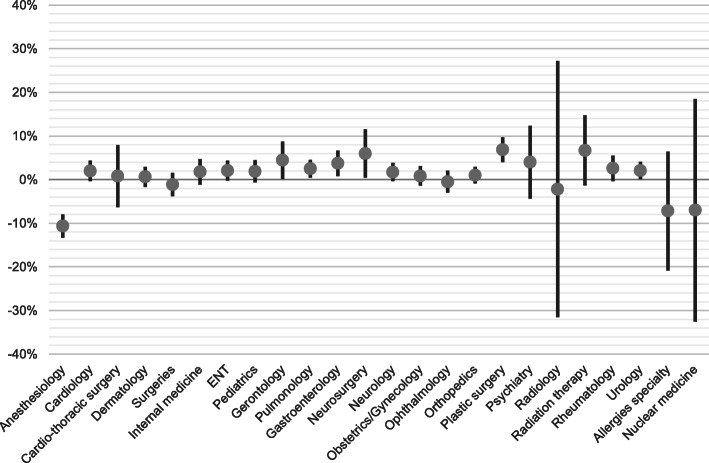
Trend annual growth in deployment of medical specialists per weighted admission, 2007–2015

Each specialty model [1] is estimated using ordinary least squares. The estimation results obtained estimating the 24 equations are presented in the Appendix. Here, we present the most important results. Figure 1 shows the point estimates of the annual growth per specialty and the corresponding 95% confidence intervals.

The findings in Fig. 1 show that the use in FTEs per weighted admission—after correction for the growth of production—increases annually in most specialties, sometimes substantially. This result implies that, over time, the input of medical specialists per admission has increased. The caveat is that the bandwidths are quite large. In several specialties, there is no strong statistical evidence that the outcome differs significantly from zero. The specialties in which there is statistically significant evidence are pulmonary medicine, gastroenterology, neurosurgery, and plastic surgery. Nevertheless, for these specialties, the outcome is uncertain in the sense that growth may be equal to, say, 1%, but may also be as much as 5%.

The notable exception applies to anaesthesiologists. This finding for this specialty shows a marked decrease in the number of FTEs per weighted admission. There has been an average annual decrease in FTEs per weighted admission by about 10% implying growing labour productivity.

**Fig. 2:**
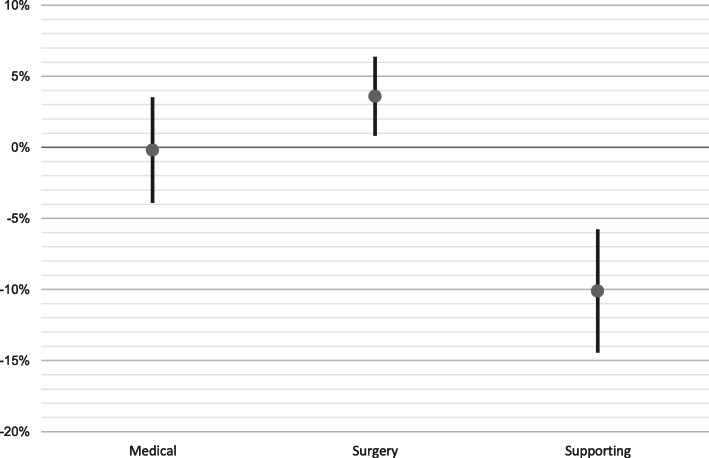
Trend annual growth in deployment of medical specialists per weighted admission, 2007–2015, per cluster

The estimates also show another interesting finding. In addition to their ‘own’ patients, many specialties also have a commitment to patients who come in through a different specialty. This applies not only to supporting specialties, but also to other specialties. In order to correct for this, we have also re-estimated the model for three clusters (surgical specialists, non-surgical specialists, and auxiliary medical staff including anaesthesiology, radiology, and pathology). The results of the estimates for clustered specialties can be found in Fig. 2. This clustering eliminates most of this mutual commitment. An important conclusion is therefore that when estimating the demand for medical specialists, one must not only look at developments in the numbers of ‘own patients’, but also consider developments in other specialties.

The growing trend in the use of medical specialists per weighted admission for medical specialties is approximately zero. However, for the surgical specialties, there is a strong growth in the admissions per FTE of about 4% per year (with a bandwidth of 0.8 to 6.3%). The number of supporting specialties faces a sharp drop of 10% per year. The latter is consistent with the results of Fig. 1, dominated by anaesthesiologists in this group. These results are clearly more robust than those of Fig. 1, due to the detailed information submitted by each specialty.

### Perceived development of labour productivity

Blank and Niaounakis [17] also present the outcomes of Delphi sessions at the end of 2018 by the Advisory Committee on Medical Manpower Planning in the Netherlands. These Delphi sessions were held with the scientific associations of almost all medical specialties. In these sessions, which included an average of seven specialists per specialty, the future trends for their field were requested in a structured way. The Delphi sessions were physical meetings where participants could give answers anonymously (via a web-based form), and the information was further distilled in several rounds [18]. The participants were asked about six developments, including professional developments, but also developments in the efficiency of the work process.

The specialists indicated that they had spent more time on such things as multidisciplinary consultations. In the 30 specialties surveyed, they anticipated seeing an increase in multidisciplinary consultations over the next 10 years. It was also concluded that alignment will take more time. All specialties, without exception, indicated that shared decision-making will require more time from the physician. These conclusions support the findings of the empirical part of this paper: a declining productivity of medical specialists.

## Conclusions

The literature review shows that attention has been paid to physician productivity for many decades. Changes in accountability such as using RVUs, incentive payments, use of staff and mid-level providers, and technology have been discussed with some consensus. Given the specialist-productivity literature, we have added to the study of specialist productivity in several ways. Firstly, Brownlee et al. [15] identified that too many specialists lead to more inefficiency. We have refined their conclusions further by assessing productivity per specialty. This can provide more information to decision-makers overseeing workforce policy, specifically where there are shortages or surpluses among physicians. Our findings that pulmonary medicine, gastroenterology, neurosurgery, and plastic surgery are statistically productive may indicate that these levels are sufficient given population needs. Secondly, based on this conjecture, we refer to the Dall et al. [16] study indicating that given the ageing population and increased disease severity, they reported that 14% more primary care providers, 20% more cardiologists, and 31% more vascular surgeons were required. We address this by demonstrating that there was current data showing that these groups of physicians demonstrated no productivity gain. So if Dall et al. [16] and Blank and van Hulst [2] are correct regarding the ageing population and technological progress, policy-makers should follow the trends of these types of physicians in order to refine whether the number of physicians in these specialties should be increased. Thirdly, Dall et al. [16] also suggested that neurologists may take on an increasing consultative role in the total treatment of patients. Being able to discern primary treating physicians from consultative physicians enables us to further discern the future role of neurologists and their total productivity. Fourthly, including a time trend enables us to assess the increasing or decreasing demand for medical students in certain specialties. It has been argued that using financial incentives promoting certain choices in some specialties can enable policy-makers to contain costs by enhancing labour productivity [1]. And, finally, including the hospital type in our analysis means we can provide policy-makers with better information regarding how specialists should be allocated while also accounting for non-patient care activities. As we argued above, hospital type can be a proxy for case mix severity. Therefore, certain specialties could be placed in types of hospitals in which productivity would be optimised.

We have enhanced the earlier studies by combining empirical findings with previously published qualitative results from the Delphi Model. All specialists participating in the Delphi study, without exception, indicated that shared decision-making will require more time from the specialist. Since we included hospital type to proxy patient severity, our findings support these qualitative findings so that informed workforce decisions can be based on empirical findings as well as physician input. Politically, including physician input may facilitate implementation of any workforce changes, since specialists are important stakeholders [19].Combining medical and administration leadership was also discussed as an important way to enhance physician productivity [14].

## Discussion

Despite the several strengths of this study, shortcomings still exist. These include the omission of patient demographic changes which, if included, could further substantiate the assumptions of an increasingly aged and sicker population. One may also reason that another shortcoming concerns the lack of quality indicators in the model specification, which may explain the low productivity figures. A simple glance at OECD data reveals that in the Netherlands, as in many other countries, quality has substantially risen in time. From these data, we observe that 5-year net survival for several types of cancer and 30-day mortality rates for AMI, haemorrhagic strokes, and ischaemic strokes all improved (https://stats.oecd.org/). If these indicators were included in the model productivity measures would have looked much better. However, regarding the purpose of forecasting demand for physicians, the presented model is to be preferred, since it is not to be expected that technical and medical innovations will cease to exist in the near future.

Another shortcoming is that specific markets were not included which could explain the possible input bias. Specifically, that wealthier markets have a greater level of technology in all their hospitals, so that specialists practising there could manipulate input measures to bias outcome assessment. Also, more detailed information on markets may correspond with a tendency for more (not necessarily better) use of resources as well as any supply-sensitive trends. Finally, there is a future need to include other labour inputs and non-patient care activities to more fully account for specialist productivity.

Balancing the strengths and shortcomings of this research, we have gone some way to providing decision-makers at all levels—public and private—a means of improving overall health care productivity that in turn may increase population well-being and provide overall social gains.

## Data Availability

The datasets generated and used for this study are not publicly available due to their confidential nature and were licenced only for use in the present study.

## Appendix

### Estimation results

Table 1 displays the estimate results. The table contains the estimated parameters for each specialty and associate standard errors (in brackets).

**Table 1:** Estimation demand for physicians, results per medical specialty

Specialties	*α*_0_ Constant	*b*_1_ Specialty admissions	*b*_2_ Hospital admissions	*d*_1_ General Hospital reference category	*d*_2_ dummy after 2013	*h*_1_ Trend
Anaesthesiology	1.401	− 0.015	1.015	− 0.189	0.447	− 0.106
	(0.082)	(0.024)	(0.024)	(0.051)	(0.088)	(0.014)
Cardiology	0.094	0.096	0.904	− 0.196	0.217	0.020
	(0.079)	(0.095)	(0.095)	(0.045)	(0.074)	(0.012)
Cardio-thoracic surgery	− 0.324	0.123	0.877	− 0.180	0.089	0.008
	(0.215)	(0.084)	(0.084)	(0.194)	(0.188)	(0.036)
Dermatology	− 0.560	0.029	0.971	− 0.046	0.314	0.006
	(0.078)	(0.019)	(0.019)	(0.051)	(0.079)	(0.012)
Surgeries	1.019	− 0.595	1.595	− 0.121	0.371	− 0.011
	(0.146)	(0.144)	(0.144)	(0.047)	(0.081)	(0.013)
Internal medicine	− 0.074	0.680	0.320	− 0.461	0.225	0.018
	(0.132)	(0.107)	(0.107)	(0.053)	(0.091)	(0.015)
ENT	− 0.469	0.377	0.623	− 0.179	0.185	0.021
	(0.062)	(0.077)	(0.077)	(0.044)	(0.071)	(0.012)
Paediatrics	0.225	1.507	− 0.507	− 0.215	0.069	0.019
	(0.069)	(0.092)	(0.092)	(0.047)	(0.079)	(0.013)
Gerontology	− 0.724	0.253	0.747	0.196	0.309	0.045
	(0.142)	(0.031)	(0.031)	(0.081)	(0.137)	(0.022)
Pulmonology	(0.058)	(0.052)	(0.052)	(0.039)	(0.065)	(0.010)
Gastroenterology	− 0.835	0.309	0.691	− 0.189	0.191	0.038
	(0.081)	(0.048)	(0.048)	(0.059)	(0.087)	(0.015)
Neurosurgery	− 0.655	0.607	0.393	− 0.398	0.154	0.060
	(0.176)	(0.039)	(0.039)	(0.140)	(0.170)	(0.028)
Neurology	0.045	0.372	0.628	− 0.116	0.239	0.017
	(0.062)	(0.079)	(0.079)	(0.040)	(0.066)	(0.011)
Obstetrics/Gynaecology	0.164	0.119	0.881	− 0.090	0.238	0.008
	(0.081)	(0.085)	(0.085)	(0.043)	(0.070)	(0.011)
Ophthalmology	− 0.228	0.265	0.735	− 0.028	0.386	− 0.005
	(0.070)	(0.039)	(0.039)	(0.050)	(0.082)	(0.013)
Orthopaedics	− 0.217	0.282	0.718	− 0.019	0.185	0.010
	(0.054)	(0.042)	(0.042)	(0.037)	(0.060)	(0.010)
Plastic surgery	− 0.801	0.663	0.337	− 0.226	0.115	0.069
	(0.086)	(0.042)	(0.042)	(0.061)	(0.086)	(0.015)
Psychiatry	− 0.428	0.146	0.854	− 0.320	− 0.085	0.040
	(0.298)	(0.061)	(0.061)	(0.193)	(0.301)	(0.043)
Radiology	6.022			0.568	− 0.826	− 0.022
	(0.991)			(0.862)	(0.819)	(0.150)
Radiation therapy	0.866	0.317	0.683	− 0.353	0.460	0.067
	(0.557)	(0.048)	(0.048)	(0.871)	(0.249)	(0.041)
Rheumatology	− 0.426	0.360	0.640	− 0.053	0.216	0.026
	(0.110)	(0.034)	(0.034)	(0.060)	(0.090)	(0.015)
Urology	− 0.596	0.227	0.773	− 0.087	0.168	0.021
	(0.060)	(0.058)	(0.058)	(0.043)	(0.062)	(0.01)
Allergies Specialty	1.537			0.673	1.061	− 0.072
	(0.411)			(0.315)	(0.411)	(0.07)
Nuclear medicine	2.819			− 0.807	1.127	− 0.070
	(0.743)			(0.918)	(0.768)	(0.130)

## Abbreviations

A&E: Accident and emergency
DRG: Diagnosis-related groups
EHR: Electronic health records
EJZ: Survey Annual Figures of Hospitals (*Enquête Jaarcijfers Ziekenhuizen*)
FTE: Full-time equivalents
LBZ: National Basic registration of Hospital Care (*Landelijke Basisregistratie Ziekenhuiszorg)*
RVU: Relative value unit
